# Poly[[tris­(*N*,*N*-dimethyl­formamide)(μ_4_-5-nitro­isophthalato)(μ_3_-5-nitro­isophthalato)dicobalt(II)] *N*,*N*-dimethyl­formamide monosolvate]

**DOI:** 10.1107/S1600536810035270

**Published:** 2010-09-08

**Authors:** Meng Su, Zhan-Dong Huang, Huang Sun, Guang Yang, Seik Weng Ng

**Affiliations:** a Department of Chemistry, Zhengzhou University, Zhengzhou 450001, People’s Republic of China; b Department of Chemistry, University of Malaya, 50603 Kuala Lumpur, Malaysia

## Abstract

In the polymeric title compound, [Co_2_(C_3_H_7_NO)_3_(C_8_H_3_NO_6_)_2_]·C_3_H_7_NO, one 5-nitro­isophthalate dianion has its two carboxyl­ate groups chelating to one Co^II^ atom while simultaneously coordinating to another metal atom in a μ_4_-bridging mode. The other 5-nitro­isophthalte dianion has one carboxyl­ate group chelating to a metal atom whereas the other bridges two metal atoms in a μ_3_-bridging mode. Both metal atoms show an octa­hedral coordination. The polymer adopts a layer motif, with the lattice dimethyl­formamide mol­ecules occupying the space between adjacent layers.

## Related literature

For adducts of cobalt 5-nitro­isophthalate, see: Chen *et al.* (2006 ▶); Du *et al.* (2008 ▶); Guo *et al.* (2006 ▶); Liu *et al.* (2008 ▶); Luo *et al.* (2003 ▶); Wang *et al.* (2008 ▶, 2009 ▶); Xie *et al.* (2006 ▶); Ye *et al.* (2008*a* ▶,*b* ▶); Yuan *et al.* (2009 ▶); Zhou *et al.* (2004 ▶).
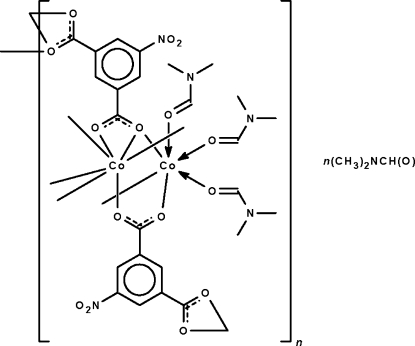


## Experimental

### 

#### Crystal data

[Co_2_(C_3_H_7_NO)_3_(C_8_H_3_NO_6_)_2_]·C_3_H_7_NO
*M* *_r_* = 828.47
Monoclinic, 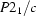

*a* = 10.0833 (12) Å
*b* = 17.0887 (19) Å
*c* = 21.074 (2) Å
β = 92.910 (2)°
*V* = 3626.7 (7) Å^3^
*Z* = 4
Mo *K*α radiation
μ = 0.99 mm^−1^
*T* = 293 K
0.40 × 0.30 × 0.30 mm

#### Data collection

Bruker SMART diffractometer
Absorption correction: multi-scan (*SADABS*; Sheldrick, 1996 ▶) *T*_min_ = 0.796, *T*_max_ = 1
21143 measured reflections
7876 independent reflections
5128 reflections with *I* > 2σ(*I*)
*R*_int_ = 0.036

#### Refinement

*R*[*F* ^2^ > 2σ(*F* ^2^)] = 0.048
*wR*(*F* ^2^) = 0.132
*S* = 0.99
7876 reflections
477 parameters
54 restraints
H-atom parameters constrained
Δρ_max_ = 0.57 e Å^−3^
Δρ_min_ = −0.45 e Å^−3^

### 

Data collection: *SMART* (Bruker, 1999 ▶); cell refinement: *SAINT* (Bruker, 1999 ▶); data reduction: *SAINT*; program(s) used to solve structure: *SHELXS97* (Sheldrick, 2008 ▶); program(s) used to refine structure: *SHELXL97* (Sheldrick, 2008 ▶); molecular graphics: *OLEX* (Dolomanov *et al.*, 2003 ▶) and *X-SEED* (Barbour, 2001 ▶); software used to prepare material for publication: *publCIF* (Westrip, 2010 ▶).

## Supplementary Material

Crystal structure: contains datablocks global, I. DOI: 10.1107/S1600536810035270/bt5344sup1.cif

Structure factors: contains datablocks I. DOI: 10.1107/S1600536810035270/bt5344Isup2.hkl

Additional supplementary materials:  crystallographic information; 3D view; checkCIF report

## Figures and Tables

**Table 1:** Selected bond lengths (Å)

Co1—O1	2.001 (2)
Co1—O3^i^	2.247 (2)
Co1—O4^i^	2.058 (2)
Co1—O7	2.383 (3)
Co1—O8	2.082 (3)
Co1—O10^ii^	2.008 (2)
Co2—O2	2.042 (2)
Co2—O7	2.091 (2)
Co2—O9^ii^	2.082 (2)
Co2—O13	2.139 (3)
Co2—O14	2.100 (2)
Co2—O15	2.063 (2)

Symmetry codes: (i) 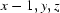; (ii) 
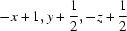
.

**Fig. 1.**
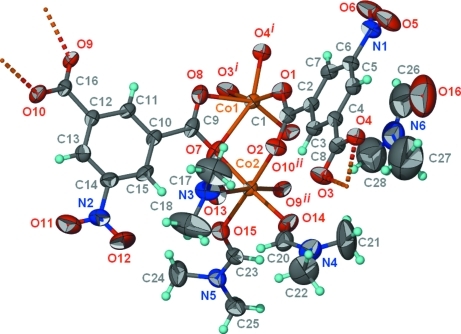
Anisotropic displacement ellipsoid plot (Barbour, 2001) of a portion of the layer structure of Co2(DMF)3(C8H3NO6)2.DMF at the 50% probability level; hydrogen atoms are drawn as spheres of arbitrary radius. Symmetry codes are given in Table 1.

## supplementary crystallographic information

### Comment

Cobalt 5-nitroisophthalate forms a number of adducts with neutral ligands (Chen *et al.,* 2006; Du *et al.*, 2008; Guo *et al.*, 2006; Liu *et al.*, 2008; Luo *et al.*, 2003; Wang *et al.*, 2008; Wang *et al.*, 2009; Xie *et al.*, 2006; Ye *et al.*, 2008*a*; Ye *et al.*, 2008*b*; Yuan *et al.*, 2009; Zhou *et al.*, 2004). The structure of the parent coordination polymer has not been reported. The attempt to synthesize this compound by using DMF as the solvent gave instead the DMF-coordinated polymer, who crystallizes as a DMF solvate (Scheme I).

In polymeric Co_2_(DFM)_3_(C_8_H_3_NO_6_)_2_^.^DMF, the 5-nitroisophthalate units show different binding modes. With one dianion, each carboxyl –CO_2_ fragment chelate to one cobalt(II) atom while simultaneously coordinating to another metal atom, *i.e.*, this dianion functions in a µ_4_-bridging mode. With the other, one carboxyl fragment chelates to a metal atom whereas the other bridges two metal atoms, the dianion functioning in a µ_3_-bridging mode. Both metal atoms show octahedral coordination. Of the two indepent metal atoms, one is coordinated by three DMF molecules. The bridging mode exercised by the dianion gives rise to a layer motif (Fig. 1). The lattice DMF molecules occupy the space between adjacent layers.

### Experimental

Cobalt(II) nitrate hexahydrate (0.5 mmol, 0.146 g) and 5-nitroisophthalic acid (0.5 mmol, 0.106 g) were dissolved in DMF (2 ml); triethylamine was allowed to diffuse into the solution. Purple crystals were obtained in 30% yield after one week.

### Refinement

H-atoms were placed in calculated positions [C–H 0.93–0.96 Å, *U*(H) 1.2–1.5*U*(C)] and were included in the refinement in the riding model approximation.

For the coordinated and lattice DMF molecules, the C–O distance was restrained to 1.25±0.01 Å, the *N*–C_carbonyl_ distance to 1.35±0.01 and the *N*–C_methyl_ distances to 1.45±0.01 Å; the molecule was restrained to lie on a plane to within 0.01 Å. For the lattice DMF molecule, the anisotropic displacement parameters of the non-H atoms were restrained to be nearly isotropic.

### Crystal data

**Table:**

[Co_2_(C_3_H_7_NO)_3_(C_8_H_3_NO_6_)_2_]·C_3_H_7_NO	*F*(000) = 1704
*M**_r_* = 828.47	*D*_x_ = 1.517 Mg m^−^^3^
Monoclinic, *P*2_1_/*c*	Mo *K*α radiation, λ = 0.71073 Å
Hall symbol: -P 2ybc	Cell parameters from 1014 reflections
*a* = 10.0833 (12) Å	θ = 2.3–26.7°
*b* = 17.0887 (19) Å	µ = 0.99 mm^−^^1^
*c* = 21.074 (2) Å	*T* = 293 K
β = 92.910 (2)°	Block, purple
*V* = 3626.7 (7) Å^3^	0.40 × 0.30 × 0.30 mm
*Z* = 4	

### Data collection

**Table:**

Bruker SMART diffractometer	7876 independent reflections
Radiation source: fine-focus sealed tube	5128 reflections with *I* > 2σ(*I*)
graphite	*R*_int_ = 0.036
ω scans	θ_max_ = 27.0°, θ_min_ = 2.0°
Absorption correction: multi-scan (*SADABS*; Sheldrick, 1996)	*h* = −12→11
*T*_min_ = 0.796, *T*_max_ = 1	*k* = −21→20
21143 measured reflections	*l* = −24→26

### Refinement

**Table:**

Refinement on *F*^2^	Primary atom site location: structure-invariant direct methods
Least-squares matrix: full	Secondary atom site location: difference Fourier map
*R*[*F*^2^ > 2σ(*F*^2^)] = 0.048	Hydrogen site location: inferred from neighbouring sites
*wR*(*F*^2^) = 0.132	H-atom parameters constrained
*S* = 0.99	*w* = 1/[σ^2^(*F*_o_^2^) + (0.0741*P*)^2^ + 0.1674*P*] where *P* = (*F*_o_^2^ + 2*F*_c_^2^)/3
7876 reflections	(Δ/σ)_max_ = 0.001
477 parameters	Δρ_max_ = 0.57 e Å^−^^3^
54 restraints	Δρ_min_ = −0.45 e Å^−^^3^

### Fractional atomic coordinates and isotropic or equivalent isotropic displacement parameters (Å^2^)

**Table:**

	*x*	*y*	*z*	*U*_iso_*/*U*_eq_	
Co1	0.54070 (4)	0.62850 (2)	0.36883 (2)	0.03535 (14)	
Co2	0.75875 (4)	0.69413 (2)	0.25276 (2)	0.03577 (14)	
O1	0.7161 (2)	0.61369 (15)	0.41609 (12)	0.0509 (6)	
O2	0.8391 (2)	0.65261 (16)	0.33703 (12)	0.0536 (7)	
O3	1.3188 (2)	0.63449 (18)	0.35333 (13)	0.0615 (8)	
O4	1.4234 (2)	0.60440 (14)	0.44350 (11)	0.0452 (6)	
O5	1.1571 (3)	0.5635 (2)	0.63158 (14)	0.0841 (10)	
O6	0.9466 (3)	0.5792 (2)	0.62357 (14)	0.0890 (11)	
O7	0.5916 (2)	0.62286 (13)	0.25975 (14)	0.0584 (7)	
O8	0.5266 (3)	0.52567 (16)	0.31596 (14)	0.0641 (8)	
O9	0.3476 (2)	0.28716 (13)	0.21118 (12)	0.0447 (6)	
O10	0.4601 (3)	0.24599 (14)	0.12880 (13)	0.0548 (7)	
O11	0.6418 (4)	0.4515 (2)	−0.00474 (17)	0.0982 (12)	
O12	0.7008 (6)	0.5587 (3)	0.0369 (2)	0.160 (2)	
O13	0.8704 (3)	0.60593 (15)	0.20713 (13)	0.0634 (8)	
O14	0.9230 (3)	0.76957 (15)	0.25177 (13)	0.0619 (7)	
O15	0.6900 (3)	0.72766 (15)	0.16294 (12)	0.0599 (7)	
O16	1.0622 (10)	0.7746 (4)	0.5743 (4)	0.236 (4)	
N1	1.0547 (3)	0.5774 (2)	0.60062 (15)	0.0565 (8)	
N2	0.6509 (4)	0.4948 (3)	0.03991 (19)	0.0781 (11)	
N3	1.0517 (3)	0.53017 (18)	0.20161 (17)	0.0715 (11)	
N4	1.1460 (3)	0.7832 (2)	0.24308 (18)	0.0788 (11)	
N5	0.6043 (3)	0.82156 (18)	0.09718 (13)	0.0572 (9)	
N6	0.9621 (7)	0.8134 (3)	0.4885 (3)	0.1170 (18)	
C1	0.8239 (3)	0.62893 (19)	0.39168 (17)	0.0385 (8)	
C2	0.9490 (3)	0.61717 (18)	0.43297 (16)	0.0361 (7)	
C3	1.0713 (3)	0.62258 (19)	0.40590 (16)	0.0390 (8)	
H3	1.0752	0.6346	0.3630	0.047*	
C4	1.1879 (3)	0.6102 (2)	0.44235 (16)	0.0381 (8)	
C5	1.1835 (3)	0.5940 (2)	0.50582 (16)	0.0411 (8)	
H5	1.2612	0.5851	0.5305	0.049*	
C6	1.0611 (3)	0.5913 (2)	0.53248 (16)	0.0399 (8)	
C7	0.9434 (3)	0.60096 (19)	0.49684 (16)	0.0394 (8)	
H7	0.8621	0.5966	0.5155	0.047*	
C8	1.3176 (3)	0.6162 (2)	0.41043 (18)	0.0411 (8)	
C9	0.5582 (3)	0.5522 (2)	0.2641 (2)	0.0454 (9)	
C10	0.5511 (3)	0.50093 (19)	0.20667 (17)	0.0400 (8)	
C11	0.4916 (3)	0.42749 (18)	0.21119 (16)	0.0389 (8)	
H11	0.4562	0.4127	0.2493	0.047*	
C12	0.4843 (3)	0.37624 (18)	0.16031 (16)	0.0374 (7)	
C13	0.5376 (3)	0.3984 (2)	0.10396 (17)	0.0458 (9)	
H13	0.5358	0.3646	0.0694	0.055*	
C14	0.5937 (4)	0.4717 (2)	0.10006 (18)	0.0525 (10)	
C15	0.6007 (4)	0.5237 (2)	0.15018 (18)	0.0509 (9)	
H15	0.6382	0.5730	0.1458	0.061*	
C16	0.4250 (3)	0.29629 (19)	0.16771 (17)	0.0391 (8)	
C17	0.9538 (4)	0.5620 (2)	0.2306 (2)	0.0616 (11)	
H17	0.9474	0.5499	0.2734	0.074*	
C18	1.0674 (8)	0.5465 (4)	0.1361 (3)	0.180 (4)	
H18A	1.0040	0.5855	0.1219	0.269*	
H18B	1.0532	0.4996	0.1117	0.269*	
H18C	1.1556	0.5656	0.1306	0.269*	
C19	1.1493 (5)	0.4793 (3)	0.2333 (3)	0.132 (3)	
H19A	1.1219	0.4670	0.2751	0.198*	
H19B	1.2337	0.5054	0.2365	0.198*	
H19C	1.1569	0.4319	0.2092	0.198*	
C20	1.0260 (4)	0.7575 (2)	0.2246 (2)	0.0653 (11)	
H20	1.0193	0.7276	0.1877	0.078*	
C21	1.1625 (6)	0.8289 (5)	0.2992 (3)	0.167 (4)	
H21A	1.0866	0.8224	0.3244	0.250*	
H21B	1.1710	0.8831	0.2880	0.250*	
H21C	1.2409	0.8123	0.3232	0.250*	
C22	1.2638 (5)	0.7666 (4)	0.2097 (3)	0.123 (2)	
H22A	1.2399	0.7391	0.1711	0.184*	
H22B	1.3228	0.7349	0.2360	0.184*	
H22C	1.3071	0.8148	0.1999	0.184*	
C23	0.6784 (3)	0.7969 (2)	0.14649 (15)	0.0503 (9)	
H23	0.7252	0.8342	0.1707	0.060*	
C24	0.5276 (6)	0.7661 (3)	0.0572 (2)	0.099 (2)	
H24A	0.5459	0.7138	0.0716	0.149*	
H24B	0.5520	0.7713	0.0139	0.149*	
H24C	0.4346	0.7769	0.0597	0.149*	
C25	0.5940 (4)	0.9038 (2)	0.08090 (19)	0.0641 (11)	
H25A	0.6564	0.9332	0.1073	0.096*	
H25B	0.5056	0.9218	0.0875	0.096*	
H25C	0.6132	0.9108	0.0371	0.096*	
C26	0.9403 (11)	0.7817 (4)	0.5443 (5)	0.175 (4)	
H26	0.8589	0.7673	0.5596	0.210*	
C27	1.0928 (11)	0.8320 (7)	0.4728 (6)	0.267 (6)	
H27A	1.1542	0.8143	0.5061	0.400*	
H27B	1.1009	0.8876	0.4680	0.400*	
H27C	1.1123	0.8066	0.4337	0.400*	
C28	0.8669 (10)	0.8325 (5)	0.4374 (4)	0.176 (4)	
H28A	0.7826	0.8096	0.4456	0.265*	
H28B	0.8972	0.8124	0.3981	0.265*	
H28C	0.8577	0.8884	0.4344	0.265*	

### Atomic displacement parameters (Å^2^)

**Table:**

	*U*^11^	*U*^22^	*U*^33^	*U*^12^	*U*^13^	*U*^23^
Co1	0.0234 (2)	0.0368 (2)	0.0459 (3)	−0.00044 (18)	0.00267 (17)	0.0023 (2)
Co2	0.0301 (2)	0.0324 (2)	0.0445 (3)	0.00062 (18)	−0.00140 (18)	0.00308 (19)
O1	0.0218 (12)	0.0657 (16)	0.0653 (17)	−0.0010 (11)	0.0021 (11)	0.0089 (13)
O2	0.0333 (14)	0.0776 (18)	0.0495 (16)	0.0072 (13)	−0.0015 (11)	0.0134 (14)
O3	0.0308 (14)	0.105 (2)	0.0496 (17)	−0.0015 (14)	0.0063 (11)	0.0135 (15)
O4	0.0236 (12)	0.0602 (15)	0.0515 (15)	0.0024 (11)	0.0001 (10)	0.0052 (12)
O5	0.073 (2)	0.128 (3)	0.0500 (18)	0.019 (2)	−0.0080 (15)	0.0166 (18)
O6	0.066 (2)	0.144 (3)	0.059 (2)	0.004 (2)	0.0244 (16)	0.0161 (19)
O7	0.0414 (15)	0.0348 (13)	0.098 (2)	−0.0069 (11)	−0.0058 (14)	−0.0051 (13)
O8	0.075 (2)	0.0554 (17)	0.0625 (19)	−0.0133 (15)	0.0114 (15)	−0.0148 (14)
O9	0.0435 (14)	0.0315 (12)	0.0598 (16)	−0.0068 (10)	0.0106 (12)	−0.0034 (11)
O10	0.0599 (17)	0.0379 (14)	0.0683 (18)	−0.0077 (12)	0.0183 (13)	−0.0083 (13)
O11	0.128 (3)	0.104 (3)	0.066 (2)	−0.006 (2)	0.035 (2)	0.008 (2)
O12	0.243 (6)	0.148 (4)	0.096 (3)	−0.123 (4)	0.055 (3)	0.006 (3)
O13	0.0591 (18)	0.0579 (16)	0.0722 (19)	0.0208 (14)	−0.0057 (14)	−0.0053 (14)
O14	0.0412 (15)	0.0610 (17)	0.085 (2)	−0.0156 (13)	0.0186 (14)	−0.0011 (15)
O15	0.080 (2)	0.0464 (15)	0.0516 (16)	0.0092 (14)	−0.0116 (14)	0.0047 (13)
O16	0.329 (8)	0.147 (5)	0.223 (6)	0.002 (6)	−0.068 (6)	−0.057 (5)
N1	0.054 (2)	0.070 (2)	0.046 (2)	0.0064 (17)	0.0045 (17)	0.0046 (16)
N2	0.087 (3)	0.089 (3)	0.059 (3)	−0.026 (2)	0.013 (2)	0.011 (2)
N3	0.072 (3)	0.068 (2)	0.077 (3)	0.031 (2)	0.028 (2)	0.009 (2)
N4	0.045 (2)	0.097 (3)	0.095 (3)	−0.014 (2)	0.008 (2)	0.003 (2)
N5	0.067 (2)	0.0534 (19)	0.0494 (19)	0.0082 (17)	−0.0100 (16)	0.0036 (15)
N6	0.134 (5)	0.098 (4)	0.120 (4)	−0.012 (3)	0.020 (4)	−0.034 (3)
C1	0.0256 (17)	0.0405 (18)	0.049 (2)	0.0047 (14)	0.0006 (14)	0.0002 (16)
C2	0.0226 (15)	0.0384 (17)	0.047 (2)	0.0009 (13)	0.0020 (13)	0.0032 (15)
C3	0.0296 (17)	0.0478 (19)	0.0393 (19)	−0.0016 (15)	0.0001 (14)	0.0044 (15)
C4	0.0255 (16)	0.0474 (19)	0.041 (2)	−0.0011 (14)	0.0003 (13)	0.0005 (15)
C5	0.0293 (17)	0.0479 (19)	0.046 (2)	0.0005 (15)	−0.0047 (14)	−0.0006 (16)
C6	0.0349 (18)	0.0449 (19)	0.0397 (19)	0.0038 (15)	0.0018 (14)	0.0041 (15)
C7	0.0262 (17)	0.0438 (18)	0.049 (2)	0.0032 (14)	0.0089 (14)	0.0034 (16)
C8	0.0228 (17)	0.049 (2)	0.052 (2)	−0.0035 (14)	0.0025 (15)	0.0001 (17)
C9	0.0286 (18)	0.038 (2)	0.069 (3)	−0.0006 (15)	−0.0030 (17)	−0.0078 (18)
C10	0.0320 (18)	0.0347 (17)	0.053 (2)	−0.0024 (14)	0.0014 (15)	0.0011 (16)
C11	0.0298 (17)	0.0367 (18)	0.050 (2)	−0.0007 (14)	0.0031 (14)	0.0028 (15)
C12	0.0287 (16)	0.0349 (17)	0.048 (2)	0.0004 (14)	−0.0018 (14)	−0.0023 (15)
C13	0.043 (2)	0.047 (2)	0.047 (2)	−0.0009 (17)	−0.0013 (16)	−0.0033 (17)
C14	0.050 (2)	0.057 (2)	0.051 (2)	−0.0101 (19)	0.0047 (18)	0.0104 (19)
C15	0.049 (2)	0.0386 (19)	0.065 (3)	−0.0122 (17)	−0.0025 (18)	0.0105 (18)
C16	0.0316 (18)	0.0357 (18)	0.049 (2)	−0.0005 (14)	−0.0046 (15)	−0.0023 (16)
C17	0.062 (3)	0.062 (3)	0.061 (3)	0.009 (2)	0.009 (2)	0.006 (2)
C18	0.281 (11)	0.150 (7)	0.117 (6)	0.123 (7)	0.099 (6)	0.046 (5)
C19	0.105 (5)	0.165 (6)	0.128 (5)	0.084 (5)	0.029 (4)	0.041 (5)
C20	0.051 (3)	0.071 (3)	0.073 (3)	−0.015 (2)	0.001 (2)	−0.007 (2)
C21	0.084 (5)	0.256 (10)	0.159 (7)	−0.058 (6)	−0.004 (4)	−0.110 (7)
C22	0.049 (3)	0.177 (7)	0.146 (6)	−0.008 (4)	0.026 (3)	−0.008 (5)
C23	0.054 (2)	0.054 (2)	0.043 (2)	0.0032 (19)	0.0003 (17)	0.0010 (18)
C24	0.136 (5)	0.072 (3)	0.085 (4)	0.001 (3)	−0.055 (3)	−0.002 (3)
C25	0.076 (3)	0.059 (3)	0.057 (3)	0.015 (2)	−0.005 (2)	0.009 (2)
C26	0.238 (9)	0.122 (6)	0.169 (7)	−0.015 (6)	0.044 (7)	−0.062 (6)
C27	0.241 (10)	0.278 (10)	0.282 (10)	−0.034 (8)	0.011 (8)	−0.061 (8)
C28	0.197 (8)	0.158 (6)	0.171 (7)	0.023 (6)	−0.014 (6)	−0.016 (6)

### Geometric parameters (Å, °)

**Table:**

Co1—O1	2.001 (2)	C2—C3	1.388 (4)
Co1—O3^i^	2.247 (2)	C3—C4	1.387 (4)
Co1—O4^i^	2.058 (2)	C3—H3	0.9300
Co1—O7	2.383 (3)	C4—C5	1.369 (5)
Co1—C8^i^	2.465 (3)	C4—C8	1.504 (4)
Co1—O8	2.082 (3)	C5—C6	1.383 (5)
Co1—O10^ii^	2.008 (2)	C5—H5	0.9300
Co2—O2	2.042 (2)	C6—C7	1.382 (4)
Co2—O7	2.091 (2)	C7—H7	0.9300
Co2—O9^ii^	2.082 (2)	C8—Co1^iii^	2.465 (3)
Co2—O13	2.139 (3)	C9—C10	1.493 (5)
Co2—O14	2.100 (2)	C10—C15	1.371 (5)
Co2—O15	2.063 (2)	C10—C11	1.396 (4)
O1—C1	1.253 (4)	C11—C12	1.383 (5)
O2—C1	1.238 (4)	C11—H11	0.9300
O3—C8	1.244 (4)	C12—C13	1.381 (5)
O3—Co1^iii^	2.247 (2)	C12—C16	1.503 (4)
O4—C8	1.260 (4)	C13—C14	1.379 (5)
O4—Co1^iii^	2.058 (2)	C13—H13	0.9300
O5—N1	1.216 (4)	C14—C15	1.379 (5)
O6—N1	1.215 (4)	C15—H15	0.9300
O7—C9	1.259 (4)	C17—H17	0.9300
O8—C9	1.240 (4)	C18—H18A	0.9600
O9—C16	1.243 (4)	C18—H18B	0.9600
O9—Co2^iv^	2.082 (2)	C18—H18C	0.9600
O10—C16	1.251 (4)	C19—H19A	0.9600
O10—Co1^iv^	2.008 (2)	C19—H19B	0.9600
O11—N2	1.196 (5)	C19—H19C	0.9600
O12—N2	1.206 (5)	C20—H20	0.9300
O13—C17	1.214 (4)	C21—H21A	0.9600
O14—C20	1.229 (4)	C21—H21B	0.9600
O15—C23	1.237 (4)	C21—H21C	0.9600
O16—C26	1.359 (8)	C22—H22A	0.9600
N1—C6	1.460 (5)	C22—H22B	0.9600
N2—C14	1.473 (5)	C22—H22C	0.9600
N3—C17	1.305 (5)	C23—H23	0.9300
N3—C18	1.426 (6)	C24—H24A	0.9600
N3—C19	1.450 (5)	C24—H24B	0.9600
N4—C20	1.326 (5)	C24—H24C	0.9600
N4—C21	1.421 (6)	C25—H25A	0.9600
N4—C22	1.439 (5)	C25—H25B	0.9600
N5—C23	1.318 (4)	C25—H25C	0.9600
N5—C25	1.449 (4)	C26—H26	0.9300
N5—C24	1.463 (5)	C27—H27A	0.9600
N6—C26	1.322 (8)	C27—H27B	0.9600
N6—C27	1.411 (8)	C27—H27C	0.9600
N6—C28	1.444 (7)	C28—H28A	0.9600
C1—C2	1.509 (4)	C28—H28B	0.9600
C2—C7	1.378 (5)	C28—H28C	0.9600
			
O1—Co1—O10^ii^	96.82 (11)	O3—C8—C4	120.1 (3)
O1—Co1—O4^i^	97.00 (10)	O4—C8—C4	118.2 (3)
O10^ii^—Co1—O4^i^	100.24 (10)	O3—C8—Co1^iii^	65.20 (18)
O1—Co1—O8	101.20 (11)	O4—C8—Co1^iii^	56.52 (16)
O10^ii^—Co1—O8	148.98 (12)	C4—C8—Co1^iii^	174.2 (3)
O4^i^—Co1—O8	102.36 (11)	O8—C9—O7	119.8 (4)
O1—Co1—O3^i^	157.87 (10)	O8—C9—C10	119.8 (3)
O10^ii^—Co1—O3^i^	87.29 (11)	O7—C9—C10	120.4 (4)
O4^i^—Co1—O3^i^	60.88 (9)	C15—C10—C11	119.5 (3)
O8—Co1—O3^i^	85.42 (12)	C15—C10—C9	122.2 (3)
O1—Co1—O7	104.29 (10)	C11—C10—C9	118.4 (3)
O10^ii^—Co1—O7	93.78 (9)	C12—C11—C10	121.5 (3)
O4^i^—Co1—O7	152.88 (9)	C12—C11—H11	119.3
O8—Co1—O7	57.43 (10)	C10—C11—H11	119.3
O3^i^—Co1—O7	97.09 (9)	C13—C12—C11	119.0 (3)
O1—Co1—C8^i^	127.71 (11)	C13—C12—C16	120.9 (3)
O10^ii^—Co1—C8^i^	94.08 (11)	C11—C12—C16	120.1 (3)
O4^i^—Co1—C8^i^	30.72 (10)	C14—C13—C12	118.6 (3)
O8—Co1—C8^i^	94.58 (11)	C14—C13—H13	120.7
O3^i^—Co1—C8^i^	30.16 (10)	C12—C13—H13	120.7
O7—Co1—C8^i^	125.81 (10)	C13—C14—C15	123.0 (3)
O2—Co2—O15	173.94 (11)	C13—C14—N2	118.3 (4)
O2—Co2—O9^ii^	97.87 (10)	C15—C14—N2	118.7 (4)
O15—Co2—O9^ii^	88.15 (10)	C10—C15—C14	118.3 (3)
O2—Co2—O7	91.26 (11)	C10—C15—H15	120.8
O15—Co2—O7	89.54 (11)	C14—C15—H15	120.8
O9^ii^—Co2—O7	89.27 (10)	O9—C16—O10	127.3 (3)
O2—Co2—O14	86.77 (11)	O9—C16—C12	117.3 (3)
O15—Co2—O14	92.79 (11)	O10—C16—C12	115.4 (3)
O9^ii^—Co2—O14	87.47 (10)	O13—C17—N3	126.3 (4)
O7—Co2—O14	175.92 (11)	O13—C17—H17	116.9
O2—Co2—O13	87.22 (11)	N3—C17—H17	116.9
O15—Co2—O13	86.74 (10)	N3—C18—H18A	109.5
O9^ii^—Co2—O13	174.03 (10)	N3—C18—H18B	109.5
O7—Co2—O13	93.76 (11)	H18A—C18—H18B	109.5
O14—Co2—O13	89.71 (11)	N3—C18—H18C	109.5
C1—O1—Co1	122.2 (2)	H18A—C18—H18C	109.5
C1—O2—Co2	149.4 (2)	H18B—C18—H18C	109.5
C8—O3—Co1^iii^	84.64 (19)	N3—C19—H19A	109.5
C8—O4—Co1^iii^	92.8 (2)	N3—C19—H19B	109.5
C9—O7—Co2	141.6 (2)	H19A—C19—H19B	109.5
C9—O7—Co1	84.1 (2)	N3—C19—H19C	109.5
Co2—O7—Co1	104.97 (11)	H19A—C19—H19C	109.5
C9—O8—Co1	98.6 (2)	H19B—C19—H19C	109.5
C16—O9—Co2^iv^	136.0 (2)	O14—C20—N4	126.0 (4)
C16—O10—Co1^iv^	132.0 (2)	O14—C20—H20	117.0
C17—O13—Co2	128.5 (3)	N4—C20—H20	117.0
C20—O14—Co2	126.0 (3)	N4—C21—H21A	109.5
C23—O15—Co2	123.1 (2)	N4—C21—H21B	109.5
O6—N1—O5	123.1 (4)	H21A—C21—H21B	109.5
O6—N1—C6	118.2 (3)	N4—C21—H21C	109.5
O5—N1—C6	118.7 (3)	H21A—C21—H21C	109.5
O11—N2—O12	122.3 (4)	H21B—C21—H21C	109.5
O11—N2—C14	119.6 (4)	N4—C22—H22A	109.5
O12—N2—C14	118.1 (4)	N4—C22—H22B	109.5
C17—N3—C18	119.7 (4)	H22A—C22—H22B	109.5
C17—N3—C19	123.1 (4)	N4—C22—H22C	109.5
C18—N3—C19	117.2 (4)	H22A—C22—H22C	109.5
C20—N4—C21	119.5 (4)	H22B—C22—H22C	109.5
C20—N4—C22	123.7 (4)	O15—C23—N5	124.7 (4)
C21—N4—C22	116.8 (5)	O15—C23—H23	117.6
C23—N5—C25	121.9 (3)	N5—C23—H23	117.6
C23—N5—C24	120.6 (3)	N5—C24—H24A	109.5
C25—N5—C24	117.5 (3)	N5—C24—H24B	109.5
C26—N6—C27	120.0 (9)	H24A—C24—H24B	109.5
C26—N6—C28	128.5 (9)	N5—C24—H24C	109.5
C27—N6—C28	111.6 (9)	H24A—C24—H24C	109.5
O2—C1—O1	126.9 (3)	H24B—C24—H24C	109.5
O2—C1—C2	116.1 (3)	N5—C25—H25A	109.5
O1—C1—C2	116.9 (3)	N5—C25—H25B	109.5
C7—C2—C3	119.8 (3)	H25A—C25—H25B	109.5
C7—C2—C1	121.0 (3)	N5—C25—H25C	109.5
C3—C2—C1	119.2 (3)	H25A—C25—H25C	109.5
C4—C3—C2	120.5 (3)	H25B—C25—H25C	109.5
C4—C3—H3	119.7	N6—C26—O16	105.3 (9)
C2—C3—H3	119.7	N6—C26—H26	127.4
C5—C4—C3	120.2 (3)	O16—C26—H26	127.4
C5—C4—C8	121.5 (3)	N6—C27—H27A	109.5
C3—C4—C8	118.3 (3)	N6—C27—H27B	109.5
C4—C5—C6	118.5 (3)	H27A—C27—H27B	109.5
C4—C5—H5	120.7	N6—C27—H27C	109.5
C6—C5—H5	120.7	H27A—C27—H27C	109.5
C7—C6—C5	122.4 (3)	H27B—C27—H27C	109.5
C7—C6—N1	118.3 (3)	N6—C28—H28A	109.5
C5—C6—N1	119.3 (3)	N6—C28—H28B	109.5
C2—C7—C6	118.5 (3)	H28A—C28—H28B	109.5
C2—C7—H7	120.7	N6—C28—H28C	109.5
C6—C7—H7	120.7	H28A—C28—H28C	109.5
O3—C8—O4	121.7 (3)	H28B—C28—H28C	109.5
			
O10^ii^—Co1—O1—C1	−72.3 (3)	C4—C5—C6—C7	2.9 (5)
O4^i^—Co1—O1—C1	−173.5 (3)	C4—C5—C6—N1	−177.5 (3)
O8—Co1—O1—C1	82.4 (3)	O6—N1—C6—C7	−4.0 (5)
O3^i^—Co1—O1—C1	−171.9 (3)	O5—N1—C6—C7	175.0 (4)
O7—Co1—O1—C1	23.4 (3)	O6—N1—C6—C5	176.3 (4)
C8^i^—Co1—O1—C1	−172.8 (2)	O5—N1—C6—C5	−4.7 (5)
O9^ii^—Co2—O2—C1	53.7 (5)	C3—C2—C7—C6	0.7 (5)
O7—Co2—O2—C1	−35.7 (5)	C1—C2—C7—C6	−179.5 (3)
O14—Co2—O2—C1	140.7 (5)	C5—C6—C7—C2	−2.8 (5)
O13—Co2—O2—C1	−129.4 (5)	N1—C6—C7—C2	177.5 (3)
O2—Co2—O7—C9	−59.1 (5)	Co1^iii^—O3—C8—O4	−0.7 (3)
O15—Co2—O7—C9	114.8 (5)	Co1^iii^—O3—C8—C4	177.4 (3)
O9^ii^—Co2—O7—C9	−157.0 (5)	Co1^iii^—O4—C8—O3	0.7 (4)
O13—Co2—O7—C9	28.1 (5)	Co1^iii^—O4—C8—C4	−177.4 (3)
O2—Co2—O7—Co1	40.41 (11)	C5—C4—C8—O3	−176.8 (3)
O15—Co2—O7—Co1	−145.60 (11)	C3—C4—C8—O3	2.4 (5)
O9^ii^—Co2—O7—Co1	−57.44 (10)	C5—C4—C8—O4	1.4 (5)
O13—Co2—O7—Co1	127.70 (10)	C3—C4—C8—O4	−179.5 (3)
O1—Co1—O7—C9	96.0 (2)	Co1—O8—C9—O7	3.2 (4)
O10^ii^—Co1—O7—C9	−166.0 (2)	Co1—O8—C9—C10	−174.3 (3)
O4^i^—Co1—O7—C9	−44.6 (3)	Co2—O7—C9—O8	103.9 (5)
O8—Co1—O7—C9	1.7 (2)	Co1—O7—C9—O8	−2.8 (3)
O3^i^—Co1—O7—C9	−78.2 (2)	Co2—O7—C9—C10	−78.5 (5)
C8^i^—Co1—O7—C9	−68.2 (2)	Co1—O7—C9—C10	174.8 (3)
O1—Co1—O7—Co2	−46.01 (12)	O8—C9—C10—C15	−171.4 (3)
O10^ii^—Co1—O7—Co2	52.01 (12)	O7—C9—C10—C15	11.1 (5)
O4^i^—Co1—O7—Co2	173.32 (15)	O8—C9—C10—C11	8.6 (5)
O8—Co1—O7—Co2	−140.32 (15)	O7—C9—C10—C11	−168.9 (3)
O3^i^—Co1—O7—Co2	139.75 (11)	C15—C10—C11—C12	1.5 (5)
C8^i^—Co1—O7—Co2	149.80 (11)	C9—C10—C11—C12	−178.5 (3)
O1—Co1—O8—C9	−101.7 (2)	C10—C11—C12—C13	0.3 (5)
O10^ii^—Co1—O8—C9	22.7 (4)	C10—C11—C12—C16	177.3 (3)
O4^i^—Co1—O8—C9	158.5 (2)	C11—C12—C13—C14	−1.5 (5)
O3^i^—Co1—O8—C9	99.7 (2)	C16—C12—C13—C14	−178.5 (3)
O7—Co1—O8—C9	−1.7 (2)	C12—C13—C14—C15	0.9 (6)
C8^i^—Co1—O8—C9	128.4 (2)	C12—C13—C14—N2	179.7 (3)
O2—Co2—O13—C17	−7.4 (3)	O11—N2—C14—C13	3.7 (7)
O15—Co2—O13—C17	172.2 (3)	O12—N2—C14—C13	−179.4 (5)
O7—Co2—O13—C17	−98.5 (3)	O11—N2—C14—C15	−177.5 (4)
O14—Co2—O13—C17	79.4 (3)	O12—N2—C14—C15	−0.6 (7)
O2—Co2—O14—C20	95.1 (3)	C11—C10—C15—C14	−2.0 (5)
O15—Co2—O14—C20	−78.8 (3)	C9—C10—C15—C14	178.0 (3)
O9^ii^—Co2—O14—C20	−166.9 (3)	C13—C14—C15—C10	0.8 (6)
O13—Co2—O14—C20	7.9 (3)	N2—C14—C15—C10	−177.9 (4)
O9^ii^—Co2—O15—C23	38.7 (2)	Co2^iv^—O9—C16—O10	2.3 (6)
O7—Co2—O15—C23	128.0 (2)	Co2^iv^—O9—C16—C12	−177.6 (2)
O14—Co2—O15—C23	−48.7 (2)	C13—C12—C16—O9	−160.5 (3)
O13—Co2—O15—C23	−138.2 (2)	C11—C12—C16—O9	22.5 (5)
Co2—O2—C1—O1	8.8 (8)	C13—C12—C16—O10	19.6 (5)
Co2—O2—C1—C2	−171.4 (4)	C11—C12—C16—O10	−157.4 (3)
Co1—O1—C1—O2	−1.7 (5)	Co2—O13—C17—N3	−153.3 (3)
Co1—O1—C1—C2	178.5 (2)	C18—N3—C17—O13	0.2 (4)
O2—C1—C2—C7	171.4 (3)	C19—N3—C17—O13	178.7 (3)
O1—C1—C2—C7	−8.7 (5)	Co2—O14—C20—N4	−149.3 (3)
O2—C1—C2—C3	−8.8 (5)	C21—N4—C20—O14	0.2 (3)
O1—C1—C2—C3	171.1 (3)	C22—N4—C20—O14	179.4 (3)
C7—C2—C3—C4	1.3 (5)	Co2—O15—C23—N5	−161.2 (2)
C1—C2—C3—C4	−178.5 (3)	C25—N5—C23—O15	179.4 (3)
C2—C3—C4—C5	−1.3 (5)	C24—N5—C23—O15	0.2 (3)
C2—C3—C4—C8	179.6 (3)	C27—N6—C26—O16	−0.3 (3)
C3—C4—C5—C6	−0.7 (5)	C28—N6—C26—O16	179.0 (3)
C8—C4—C5—C6	178.4 (3)		

Symmetry codes: (i) *x*−1, *y*, *z*; (ii) −*x*+1, *y*+1/2, −*z*+1/2; (iii) *x*+1, *y*, *z*; (iv) −*x*+1, *y*−1/2, −*z*+1/2.
